# Adjuvant Chemotherapy Necessity in Stage I Ovarian Endometrioid Carcinoma: A SEER-Based Study Verified by Single-Center Data and Meta-Analysis

**DOI:** 10.32604/or.2025.065137

**Published:** 2025-09-26

**Authors:** Liang Yu, Mingrui Zhao, Jinhui Liu, Yuqin Yang, Lin Zhang, Wenjun Cheng

**Affiliations:** 1 Department of Obstetrics and Gynecology, The First Clinical Medical College of Nanjing Medical University, The First Affiliated Hospital of Nanjing Medical University, Nanjing, 210029, China; 2 Department of Gynecology, The First Affiliated Hospital of Nanjing Medical University (Jiangsu Province Hospital), Nanjing, 210029, China

**Keywords:** Endometrioid ovarian cancer, adjuvant chemotherapy, stage I, surveillance, epidemiology, and end results (SEER)

## Abstract

**Background:**

The benefit of adjuvant chemotherapy for stage I ovarian endometrioid carcinoma (OEC) remains controversial. Hence, the study sought to explore its value in stage I OEC patients.

**Methods:**

Stage I OEC patients (1988–2018) were identified from the Surveillance, Epidemiology, and End Results (SEER) database. Multivariate Cox analysis was used to control confounders. Logistic regression was used to explore factors associated with adjuvant chemotherapy. Cox regression analysis and Kaplan-Meier curves were used to assess the survival benefits. Single-center clinical data and meta-analysis following PRISMA guidelines provided external validation.

**Result:**

Adjuvant chemotherapy correlated with improved survival (Hazard Ratio (HR): 0.860, *p* = 0.011), as did lymphadenectomy (HR: 0.842, *p* < 0.001). Higher age, pathological stage, and tumor grade negatively affected survival. Chemotherapy administration associated with higher pathological stage (IB: Odds Ratio (OR) 1.565, *p* < 0.001; IC: OR 4.091, *p* < 0.001), higher grade (G2: OR 2.336, *p* < 0.001; G3: OR 4.563, *p* < 0.001), and lymphadenectomy (OR 1.148, *p* = 0.040). Stratification analysis showed adjuvant chemotherapy failed to improve prognosis in stage IA/IB patients regardless of grade or lymphadenectomy. For stage IC patients, chemotherapy benefited grade 1-2 or grade 3 patients without lymphadenectomy, and grade 3 patients with lymphadenectomy. Meta-analysis revealed reduced recurrence in stage IC patients (OR = 0.50, *p* = 0.035).

**Conclusion:**

Adjuvant chemotherapy confers survival benefits for stage IC patients, particularly those without lymphadenectomy.

## 1 Introduction

Ovarian endometrioid carcinoma (OEC) is a rare subtype, accounting for about 10% of epithelial ovarian cancers [1]. It is often well differentiated, discovered early, associated with endometriosis, and has a favorable prognosis [2]. The current therapeutic paradigm consists of primary cytoreductive surgery followed by adjuvant chemotherapy, yet the indications for chemotherapy remain controversial across international guidelines. Current consensus recommends observation for stage IA, grade 1, and adjuvant chemotherapy for stage IC, grade 3. However, the benefits of chemotherapy for stage IA grade 2-3, stage IB grade 1-3, and stage IC grade 1-2 remain controversial (National Comprehensive Cancer Network (NCCN) [3,4]; International Federation of Gynecology and Obstetrics (FIGO) [5]; Chinese Society of Clinical Oncology (CSCO) [6,7]; Korean Society of Gynecologic Oncology (KSGO) [8]; Japan Society of Gynecologic Oncology (JSGO) [9]; Spanish Society for Medical Oncology (SEOM) [10]; European Society for Medical Oncology (ESMO) [11]). Clinical studies also offer conflicting views [12,13]. To resolve this ambiguity, the study conducted a Surveillance, Epidemiology, and End Results (SEER) database analysis to evaluate the survival impact of chemotherapy in stage I OEC. Furthermore, the study integrated single-institution data with a systematic meta-analysis of prior studies to provide a comprehensive assessment, aiming to refine postoperative chemotherapy selection criteria for early-stage OEC and optimize patient outcomes.

## 2 Methods

### 2.1 Data Source and Collection in the SEER Database

The study population was derived from the Surveillance, Epidemiology, and End Results database (SEER, November 2020 data submission). The target data were downloaded by using SEER*Stat 8.3.9.2 software. Patients diagnosed with OEC were defined by the International Classification of Diseases for Oncology, third edition (ICD-O-3, https://www.who.int/standards/classifications/other-classifications/international-classification-of-diseases-for-oncology, accessed on 17 June 2025). The histology codes included: 8380/3, 8381/3, 8382/3 and 8383/3. The primary tumor site code was C56.9-Ovary. Yu Liang searched and collected data from the SEER database.

Those patients who met the following inclusion and exclusion criteria were enrolled. The inclusion criteria: (1) Patients were histologically diagnosed with OEC as the first primary tumor; (2) Patients were diagnosed between 1988 and 2018, at stage I; (3) Patients who had complete records of the following variables: (I) year of diagnosis; (II) age at diagnosis; (III) race/ethnicity; (IV) pathological stage; (V) tumor grade; (VI) surgery at primary site; (VII) chemotherapy; (VIII) survival data including survival time and vital status; (4) Patients who had at least one month of follow-up. The exclusion criteria: (1) Patients who did not undergo primary tumor-related surgery; (2) Patients with stage I not otherwise specified.

Data from the SEER is available for the public and the access license was obtained from the SEER (ID: 18177-Nov2020). Hence, institutional review board (IRB) approval was not required in this part.

### 2.2 Data Source and Collection in Our Single Center

We collected clinical data on more than 1000 ovarian cancer patients hospitalized at the First Affiliated Hospital of Nanjing Medical University between 2010 and 2021. The inclusion criteria: (1) Patients were histologically diagnosed with OEC as the only primary tumor; (2) Patients were diagnosed between 2010 and 2021, at stage I; (3) Patients who had complete records of the following variables: (I) year of diagnosis; (II) age at diagnosis; (III) residential address and contact information; (IV) pathological stage; (V) tumor grade; (VI) surgery at primary site; (VII) chemotherapy; (VIII) survival data including survival time and vital status; (X) immunohistochemical parameters: WT-1(−), ER/PR(+), TP53wt, PAX8(+), Napsin A(−); (4) Patients who had at least one month of follow-up. Patients were followed up until May 2024. Zhao Mingrui searched and collected data on ovarian cancer patients from our single center. Whether survival analysis was performed depended on the sample size and survival data. If data were limited, descriptive analysis was performed. Our single-center retrospective study was approved by the Ethics Committee of the First Affiliated Hospital of Nanjing Medical University (2020-MD-371).

### 2.3 Meta-Analysis for Verification

The part of the meta-analysis was based on the PRISMA guideline and undertaken to identify clinical trials or cohort studies concerning the effect of adjuvant chemotherapy on stage I OEC (The PRISMA checklist can be found in the supplementary files-PRISMA checklist). A literature search was performed in PubMed, EMBASE, MEDLINE, Google Scholar, Cochrane Library and CNKI, which included records up to December 2024. The keywords included “adjuvant chemotherapy”, “endometrioid ovarian cancer” and “stage I”. The inclusion criteria were as follows: (1) randomized controlled trials (RCTs) and cohort studies; (2) patients exposed to adjuvant chemotherapy or no adjuvant chemotherapy; (3) the follow-up time was long enough to demonstrate a treatment difference. The exclusion criteria were as follows: (1) Non-English or non-Chinese literature; (2) Repeated and inaccessible literature; (3) Literature with incomplete data. The Newcastle-Ottawa Scale (NOS) was used to evaluate the quality of the included studies. The main items include 3 parts: patient selection, intergroup comparability and outcome measurement. A total score of more than 6 was considered to be of satisfactory quality. Two independent researchers screened the literature, evaluated quality and extracted data independently. Any disagreements were discussed and solved by consensus or third-party arbitration. Institutional review board (IRB) approval was also not required in this part.

### 2.4 Statistical Analysis

Pearson χ^2^ tests and Mann-Whitney U tests were used to evaluate the distributions of categorical and continuous variables between patients with or without adjuvant chemotherapy. Multivariate Cox regression analysis was performed to control for potential confounders and identify independent prognostic factors associated with survival. The results were presented as hazard ratios (HR) with corresponding 95% confidence intervals (CI). The binary logistic regression was performed to identify factors linked with the administration of adjuvant chemotherapy. The survival benefit of adjuvant chemotherapy was further assessed in stratified analyses using Kaplan-Meier survival analysis [14] and Cox regression analysis [15]. All statistical analyses were performed with IBM SPSS 26.0, X-tile 3.6 and R 4.3 software.

The meta-analysis was performed using STATA 12.0. The binary variables were evaluated by odds ratio (OR) and its 95% confidence interval (95% CI). According to the heterogeneity, the appropriate model (random or fixed) was then selected to merge the outcome indicators. All in all, *p* < 0.05 was considered to indicate a statistically significant result.

## 3 Results

### 3.1 Patient Demographics

A total of 5932 eligible stage I early-onset ovarian cancer patients were identified. Follow-up duration ranged from 1 to 371 months, with a median of 101 months. Among these, 3456 patients were stage IA (58.3%), 335 were stage IB (5.6%), and 2141 were stage IC (36.1%). Most patients had grade 1 (n = 2480, 41.8%) or grade 2 (n = 2414, 40.7%) tumors, with only 1038 classified as grade 3 (17.5%). The median age was 54 years (interquartile range 18), predominantly White (84.7%), and 3790 patients (63.9%) underwent lymphadenectomy.

In total, 2779 patients received adjuvant chemotherapy, while 3153 did not. Those who received chemotherapy were younger (median age 53 vs. 55 years, *p* < 0.01). Chemotherapy use increased over time, with 43.4% and 43.5% of OEC patients receiving it during 1988–1997 and 1998–2007, respectively; this rate rose to 50.5% from 2008–2018. Furthermore, adjuvant chemotherapy use increased with tumor stage and grade: 33.3% in stage IA, 48.1% in stage IB, and 68.6% in stage IC (*p* < 0.01). The usage rate for grade 3 tumors was 67.8%, compared to 52.3% and 32.8% for grade 2 and grade 1, respectively (*p* < 0.01). Additionally, 49.3% of patients who underwent lymphadenectomy also received adjuvant chemotherapy. Demographic and clinical characteristics are summarized in Table 1.

**Table 1:** Basic characteristics of patients with stage I OEC from the SEER database

Basic characteristics		Total	No chemotherapy	Chemotherapy	*p*-value
Number of patients		5932	3153(53.2%)	2779(46.8%)	
Age at diagnosis					
	Median (IQR)	54(18)	55(20)	53(16)	<0.001
	Range	17–98	18–98	17–94	
Years of diagnosis					
	1988–1997	837(14.1%)	474(56.6%)	363(43.4%)	<0.001
	1998–2007	2215(37.3%)	1252(56.5%)	963(43.5%)	
	2008–2018	2880(48.6%)	1427(49.5%)	1453(50.5%)	
Race					
	White	5026(84.7%)	2682(53.4%)	2344(46.6%)	0.201
	Black	303(5.1%)	169(55.8%)	134(44.2%)	
	Other	603(10.2%)	302(50.1%)	301(49.9%)	
Grade					
	G1	2480(41.8%)	1667(67.2%)	813(32.8%)	<0.001
	G2	2414(40.7%)	1152(47.7%)	1262(52.3%)	
	G3	1038(17.5%)	334(32.2%)	704(67.8%)	
Stage					
	IA	3456(58.3%)	2306(66.7%)	1150(33.3%)	<0.001
	IB	335(5.6%)	174(51.9%)	161(48.1%)	
	IC	2141(36.1%)	673(31.4%)	1468(68.6%)	
Lymphadenectomy					
	No/unknown	2142(36.1%)	1231(57.5%)	911(42.5%)	<0.001
	Yes	3790(63.9%)	1922(50.7%)	1868(49.3%)	

### 3.2 Prognostic Factors Associated with the Overall Survival of OEC

In this analysis, multivariate Cox regression was employed to control for confounding factors and identify independent prognostic factors in stage I early-onset ovarian cancer patients. Median age served as a distinguishing factor in the analysis. The results indicated that adjuvant chemotherapy (HR: 0.860, 95% CI: 0.766–0.967, *p* = 0.011) and lymphadenectomy (HR: 0.842, 95% CI: 0.749–0.946, *p* < 0.001) were protective prognostic factors. Conversely, older age, later diagnosis, higher pathological stage, and tumor grade were associated with poorer prognosis. Patients over 54 years had a significantly worse prognosis (HR: 3.088, 95% CI: 2.744–3.475, *p* < 0.001). Those diagnosed in 1998–2007 (HR: 1.349, 95% CI: 1.155–1.574, *p* < 0.001) and 2008–2018 (HR: 1.301, 95% CI: 1.070–1.583, *p* = 0.008) also showed worse outcomes compared to those diagnosed in 1988–1997. Higher tumor grades were linked to deteriorating prognosis: patients with grade 2 (HR: 1.209, 95% CI: 1.064–1.375, *p* = 0.004) and grade 3 tumors (HR: 1.853, 95% CI: 1.599–2.147, *p* < 0.001) had worse outcomes compared to grade 1. Furthermore, the risk of death increased with advancing pathological stage; patients with stage IB (HR: 1.354, 95% CI: 1.099–1.667, *p* = 0.004) and stage IC (HR: 1.240, 95% CI: 1.099–1.398, *p* < 0.001) had worse survival compared to stage IA patients. Notably, race showed no significant correlation with prognosis. These findings are detailed in Table 2.

**Table 2:** Multivariate survival analyses of patients with stage I OEC

Basic characteristics		Hazard ratio	95% confidence intervals		*p-*value
			Lower	Upper	
Age at diagnosis					
	≤54	Reference			
	>54	3.088	2.744	3.475	<0.001
Years of diagnosis					
	1988–1997	Reference			
	1998–2007	1.349	1.155	1.574	<0.001
	2008–2018	1.301	1.070	1.583	0.008
Race					
	White	Reference			
	Black	1.023	0.793	1.319	0.862
	Other	1.082	0.916	1.279	0.353
Grade					
	G1	Reference			
	G2	1.209	1.064	1.375	0.004
	G3	1.853	1.599	2.147	<0.001
Stage					
	IA	Reference			
	IB	1.354	1.099	1.667	0.004
	IC	1.240	1.099	1.398	<0.001
Chemotherapy					
	NO	Reference			
	YES	0.860	0.766	0.967	0.011
Lymphadenectomy					
	No/unknown	Reference			
	Yes	0.842	0.749	0.946	<0.001

### 3.3 Predictors Linked with the Receipt of Adjuvant Chemotherapy for OEC

To identify factors associated with adjuvant chemotherapy administration, the study conducted a binary logistic regression analysis, the results of which are presented in **Table S1**. Significant factors included years of diagnosis, age at diagnosis, tumor grade, pathological stage, and lymphadenectomy performance. Advancing pathological stage and tumor grade showed strong correlations; patients in stage IB (OR: 1.565, 95% CI: 1.236–1.981, *p* < 0.001) and stage IC (OR: 4.091, 95% CI: 3.629–4.612, *p* < 0.001) were more likely to receive adjuvant chemotherapy than those in stage IA. Similarly, patients with grade 2 (OR: 2.336, 95% CI: 2.062–2.647, *p* < 0.001) and grade 3 tumors (OR: 4.563, 95% CI: 3.860–5.393, *p* < 0.001) were more likely to receive chemotherapy compared to grade 1 patients. Performance of lymphadenectomy was also positively associated with adjuvant chemotherapy (OR: 1.148, 95% CI: 1.006–1.311, *p* = 0.040). Additionally, younger patients (OR: 0.785, 95% CI: 0.701–0.880, *p* < 0.001) and those newly diagnosed (OR: 1.201, 95% CI: 1.097–1.315, *p* < 0.001) were more likely to receive adjuvant chemotherapy.

### 3.4 Current Guideline Recommendations on the Administration of Adjuvant Chemotherapy in Stage I OEC

The recommendations for adjuvant chemotherapy based on the following guidelines were summarized in **Table S2**. In conclusion, observation was commonly recommended in patients with stage IA grade 1 and adjuvant chemotherapy was necessary for patients with stage IC grade 3. This view was widely shared by current guidelines. However, whether adjuvant chemotherapy should be used in patients with stage IA grade 2-3, stage IB grade 1-3 and stage IC grade 1-2 was still controversial (NCCN [3,4]; FIGO [5]; CSCO [6,7]; KSGO [8]; JSGO [9]; SEOM [10]; ESMO [11]).

#### Stratification Survival Analysis Based on Predictors of Adjuvant Chemotherapy

To address the controversies in the current guidelines, the study performed a stratification analysis. Grade 1 and 2 patients were grouped according to multivariate Cox and binary logistic regression, and lymphadenectomy, tumor grade, and pathological stage were selected as stratification variables.

**Figure 1:**
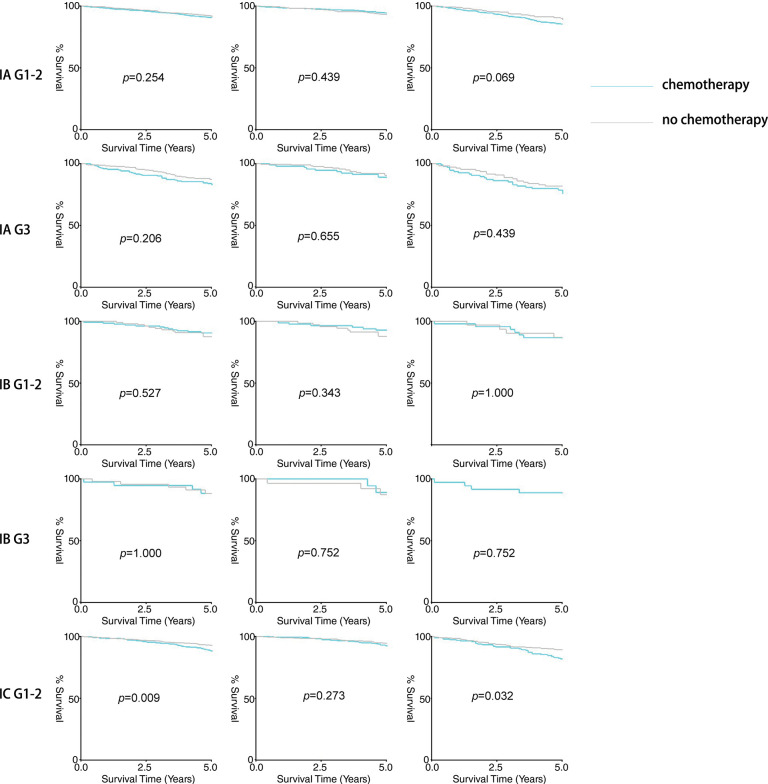

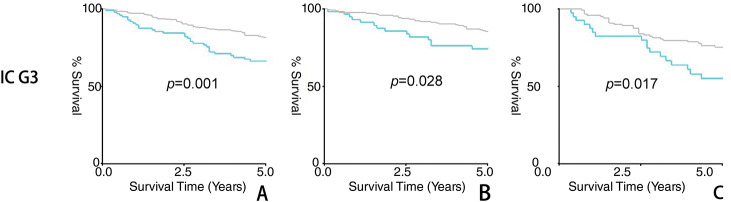
Result of five-year survival analysis between the adjuvant chemotherapy group and non-adjuvant chemotherapy group in patients with stage I OEC. (**A**): All patients; (**B**): Patients who received lymphadenectomy; (**C**): Patients who did not receive lymphadenectomy

Firstly, the study compared the 5-year overall survival (OS) rates. As shown in Table 3 and Fig. 1, no significant differences were observed in stage IA/IB patients with tumor grades 1-2 or grade 3, regardless of lymphadenectomy. However, for stage IC grade 1-2 patients, the chemotherapy group had a higher 5-year OS rate (92.8% vs. 88.4%, *p* = 0.009), and for stage IC grade 3 patients, the rate was also higher (81.6% vs. 66.3%, *p* = 0.001). In subgroup analyses, adjuvant chemotherapy improved the 5-year survival for stage IC grade 1-2 (89.2% vs. 81.8%, *p* = 0.032) and grade 3 (75.2% vs. 55.1%, *p* = 0.017) patients without lymphadenectomy, and also for grade 3 patients with lymphadenectomy (85.3% vs. 74.2%, *p* = 0.028). However, no significant difference was noted for stage IC grade 1-2 patients with lymphadenectomy (94.4% vs. 92.2%, *p* = 0.273).

**Table 3:** Five-year overall survival rates in patients with stage I ovarian endometrioid carcinoma according to adjuvant chemotherapy status, stratified by substage, tumor grade, and lymphadenectomy

Stage	Chemotherapy (**+**)			Chemotherapy (**–**)			*p*-value
	Total	n	5-Y OS rate	Total	n	5-Y OS rate	
IA G1/2	846	59	92.1%	2110	174	90.7%	0.254
IA G1/2 no LND	296	28	85.4%	830	113	89.8%	0.069
IA G1/2 with LND	550	31	93.4%	1280	61	94.4%	0.439
IA G3	304	33	87.2%	196	31	83.3%	0.206
IA G3 no LND	112	19	82.0%	96	21	77.5%	0.439
IA G3 with LND	192	14	90.5%	100	10	89.1%	0.655
IB G1/2	114	12	87.5%	136	12	90.6%	0.527
IB G1/2 no LND	34	4	86.9%	46	6	86.8%	>0.999
IB G1/2 with LND	80	8	87.8%	90	6	92.7%	0.343
IB G3	47	5	88.3%	38	4	88.4%	>0.999
IB G3 no LND	19	2	89.5%	17	2	88.2%	0.752
IB G3 with LND	28	3	87.2%	21	2	88.9%	0.752
IC G1/2	1115	66	92.8%	573	56	88.4%	**0.009**
IC G1/2 no LND	325	31	89.2%	202	33	81.8%	**0.032**
IC G1/2 with LND	790	35	94.4%	371	23	92.2%	0.273
IC G3	353	58	81.6%	100	31	66.3%	**0.001**
IC G3 no LND	125	29	75.2%	40	17	55.1%	**0.017**
IC G3 with LND	228	29	85.3%	60	14	74.2%	**0.028**

Note: LND, lymphadenectomy; Y, year; OS, overall survival. Bold *p*-values indicate statistical significance, which means they are <0.05.

**Figure 2:**
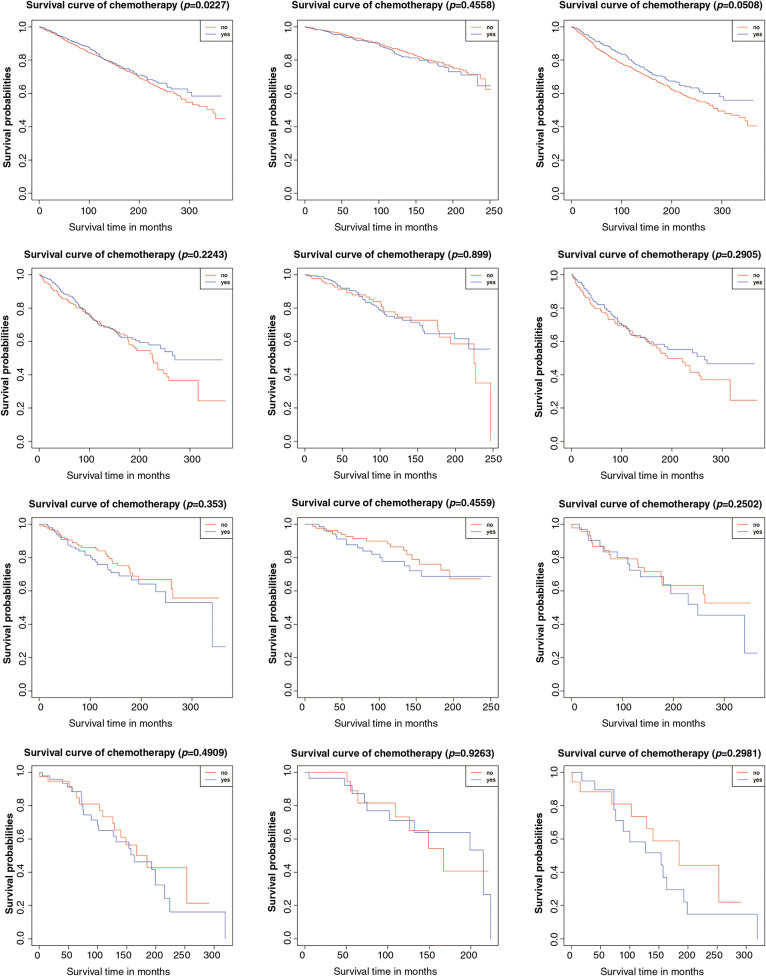

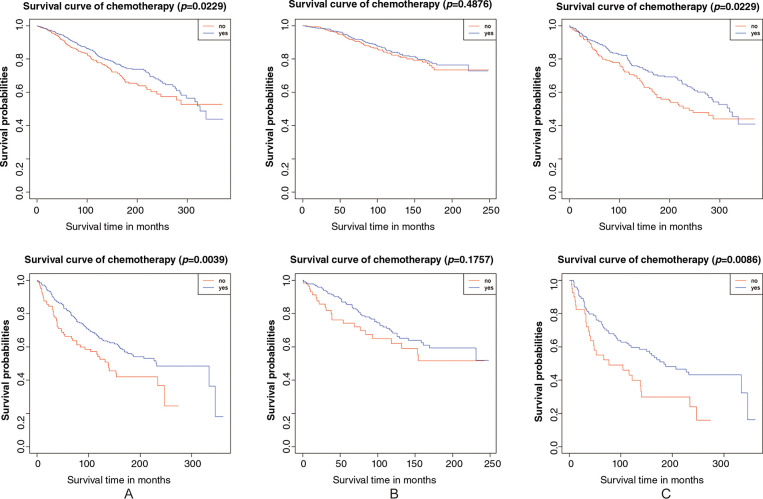
Result of overall survival analysis between the adjuvant chemotherapy group and non-adjuvant chemotherapy group in patients with stage I OEC. (**A**): All patients; (**B**): Patients who received lymphadenectomy; (**C**): Patients who did not receive lymphadenectomy

Secondly, we evaluated overall survival using hazard ratios, presented in Table 4, supported by Kaplan–Meier analysis. As previously noted, no significant differences were found in stage IA/IB patients with either tumor grade, irrespective of lymphadenectomy. In stage IC grade 1-2 (HR: 0.770, 95% CI: 0.641–0.965, *p* = 0.023) and grade 3 (HR: 0.615, 95% CI: 0.441–0.858, *p* = 0.004) subgroups, better prognoses were seen in the chemotherapy group. Further stratification indicated that chemotherapy improved prognosis for stage IC grade 1-2 (HR: 0.716, 95% CI: 0.529–0.969, *p* = 0.031) and grade 3 (HR: 0.546, 95% CI: 0.345–0.864, *p* = 0.010) patients without lymphadenectomy, but not for those with lymphadenectomy (grade 1-2 HR: 0.886, 95% CI: 0.629–1.248, *p* = 0.489; grade 3 HR: 0.715, 95% CI: 0.439–1.165, *p* = 0.178). Kaplan–Meier survival analysis results were presented in Fig. 2.

**Table 4:** Overall survival analysis of stage I OEC patients with or without adjuvant chemotherapy, stratified by sub-stage, tumor grade, and lymphadenectomy

Stage	Hazard ratio	95% confidence intervals		*p-*value
		Lower	Upper	
IA G1/2	0.894	0.747	1.070	0.223
IA G1/2 no LND	0.788	0.620	1.000	0.051
IA G1/2 with LND	1.110	0.844	1.459	0.456
IA G3	0.827	0.608	1.124	0.224
IA G3 no LND	0.807	0.542	1.202	0.292
IA G3 with LND	0.969	0.593	1.584	0.899
IB G1/2	1.264	0.771	2.074	0.354
IB G1/2 no LND	1.259	0.620	2.559	0.524
IB G1/2 with LND	1.302	0.650	2.606	0.456
IB G3	1.262	0.656	2.426	0.486
IB G3 no LND	1.599	0.659	3.881	0.296
IB G3 with LND	0.954	0.353	2.579	0.926
IC G1/2	0.770	0.614	0.965	**0.023**
IC G1/2 no LND	0.716	0.529	0.969	**0.031**
IC G1/2 with LND	0.886	0.629	1.248	0.489
IC G3	0.615	0.441	0.858	**0.004**
IC G3 no LND	0.546	0.345	0.864	**0.010**
IC G3 with LND	0.715	0.439	1.165	0.178

Note: LND, lymphadenectomy. Bold *p*-values indicate statistical significance, which. means they are <0.05.

### 3.5 Descriptive Analysis of Our Single-Center Data on the Administration of Adjuvant Chemotherapy in Stage I OEC

A total of 40 patients with stage I OEC were initially reviewed, with seven excluded due to other OC subtypes or non-ovarian primary tumors. The remaining 33 patients were included for analysis, though the cohort size precluded formal survival analysis, limiting assessment to quantitative descriptive statistics. The median patient age was 49 years (interquartile range 11). All patients underwent comprehensive staging surgery, including lymphadenectomy: 9 had stage IA disease while 24 had stage IC. Histologically, 21 patients had grade 1-2 tumors, 7 had grade 3, and 5 had unspecified grade.

Adjuvant chemotherapy was administered to 23 patients, all receiving paclitaxel-platinum regimens (cisplatin or carboplatin). The chemotherapy group comprised 3 stage IA and 20 stage IC patients, while the non-chemotherapy group included 6 stage IA and 4 stage IC patients. As of May 2024, five deaths were recorded: two in stage IA (one treated with chemotherapy, one without) and three in stage IC (one treated, two untreated). Notably, all chemotherapy-naïve patients had grade 1-2 tumors. Based on these findings, our institutional practice favors adjuvant chemotherapy for grade 3 and stage IC patients.

### 3.6 Meta-Analysis on the Administration of Adjuvant Chemotherapy in Stage I OC

**Figure 3:**
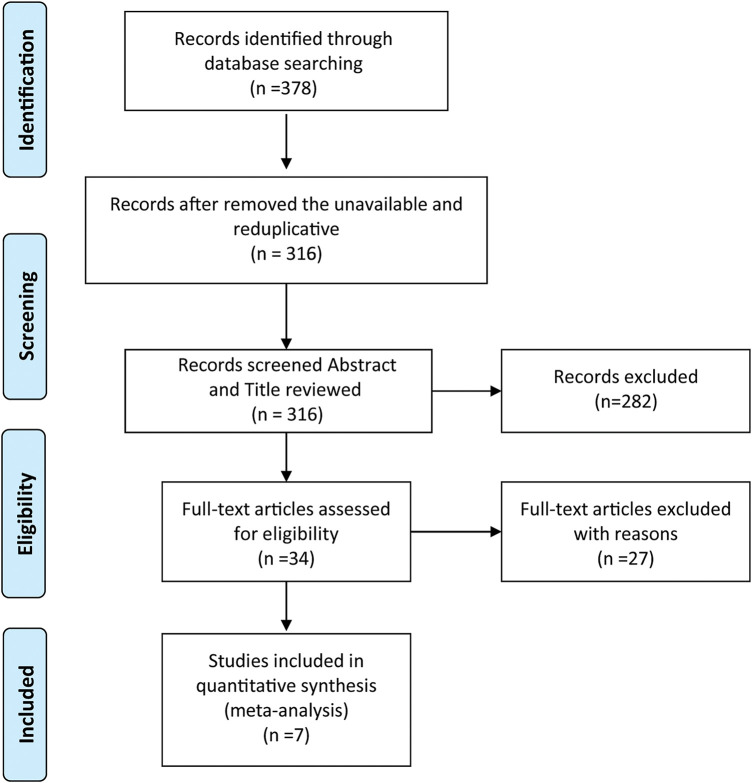
PRISMA flow chart

**Figure 4:**
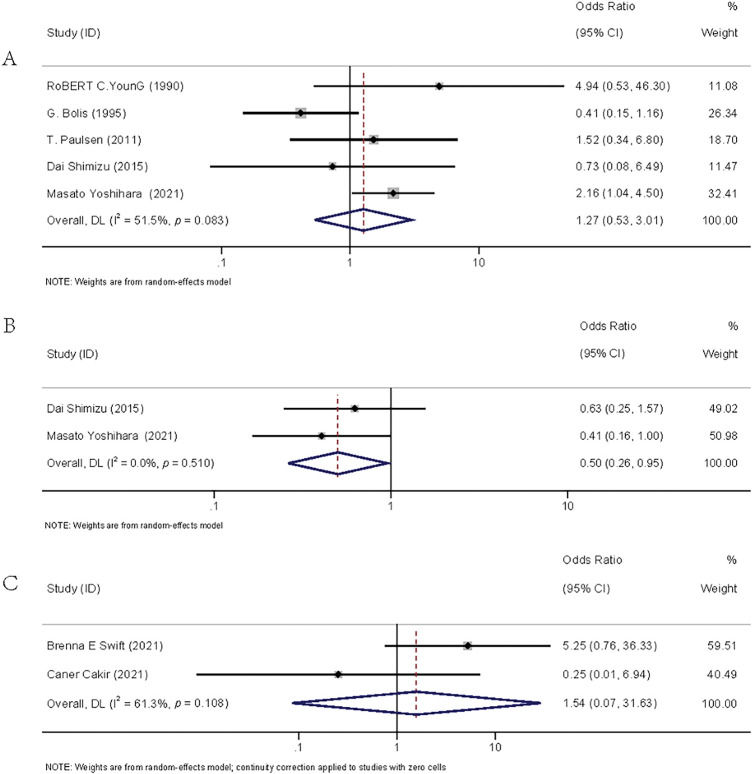
Forest plots of recurrence rates between the adjuvant chemotherapy group and the non-adjuvant chemotherapy group. (**A**): stage IA/B OC [16–19,22]; (**B**): stage IC OC [19,22]; (C): stage IA/B OEC [20–21]

The flow diagram detailing the identification and inclusion process of targeted literature is shown in Fig. 3. Seven studies were included in the final meta-analysis, spanning three distinct time periods: two from 1990–2000 [16,17], two from 2010–2020 [18,19], and three from 2021–2025 [20–22]. The basic information of the included literature was presented in **Table S3.** Meta-analyses showed that adjuvant chemotherapy did not affect the recurrence of patients with stage IA/B OC (OR = 1.27, 95% CI: 0.53–3.01, *p* = 0.595, I^2^ = 51.50%) or stage IA/B OEC (OR = 1.54, 95% CI: 0.07–31.63, *p* = 0.780, I^2^ = 61.30%), but it could reduce the recurrence rate of patients with stage IC OC (OR = 0.50, 95% CI: 0.26–0.95, *p* = 0.035, I^2^ = 0.00%). The result is given in Fig. 4.

## 4 Discussion

Firstly, in the survival analysis of stage IA/IB patients, chemotherapy did not appear to provide survival benefits for those with grade 1-2, regardless of lymphadenectomy. Additionally, the meta-analysis showed no reduction in recurrence risk from chemotherapy for stage IA/IB OEC patients vs. observation.

Second, in stage IC grade 1-2 patients, chemotherapy significantly improved both 5-year and overall survival. Among those who did not undergo lymphadenectomy, adjuvant chemotherapy demonstrated superior outcomes compared to observation. However, no survival benefit was observed in patients who received lymphadenectomy.

Third, for stage IC grade 3 patients, chemotherapy was associated with improved 5-year survival. Notably, patients without lymphadenectomy benefited in both 5-year and overall survival, whereas those who underwent lymphadenectomy only showed a 5-year survival advantage without significant overall survival improvement—a discrepancy potentially attributable to selection bias, given that half of the cases were derived from 2008-2018. Furthermore, our meta-analysis confirmed that chemotherapy significantly reduced recurrence rates in stage IC ovarian cancer patients.

This study’s first findings were consistent with those of some previous studies. Kumar et al., found no benefit in disease-free survival (DFS) for stage IA/IB OEC patients from adjuvant chemotherapy [23]. Cybulska et al., reported no progression-free survival (PFS) advantage [24], and Swift et al., also showed no survival improvement for grade 1-2 patients in stage IA/IB [20]. Our study, with a larger sample size compared to a SEER-based study, confirmed these findings [12]. A Cochrane meta-analysis on early-stage epithelial ovarian cancer also suggested withholding chemotherapy for stage IA/IB grade 1-2 patients [25]. However, some studies present opposing views. Chatterjee et al. found chemotherapy reduced mortality in stage IA/IB grade 2 patients but not in grade 1 [26]. Nasioudis et al. similarly indicated survival benefits for grade 2 patients, but none for grade 1 [13].

For grade 3 patients, no significant survival difference was observed between chemotherapy and observation, as supported by Nasioudis et al., [13] and Oseledchyk et al. [12]. However, Chatterjee et al. found chemotherapy reduced mortality in grade 3 patients [26]. The Cochrane meta-analysis also recommended chemotherapy for stage IA/IB grade 3 [25], noting that poorly differentiated tumors are more aggressive and tend to have worse prognoses [27,28].

Multiple studies supported our second result. ICON1’s extended follow-up suggested chemotherapy benefits for stage IC grade 2 [29], and Cybulska et al. found progression-free survival (PFS) benefits from chemotherapy in stage IC OEC [24]. Our results, based on a larger sample size, differ from Oseledchyk et al., who found no benefit from chemotherapy regardless of lymphadenectomy [12]. Nasioudis et al., however, reported chemotherapy benefits for stage IC grade 2 with lymphadenectomy, but not without it [13]. Nonetheless, we maintain our findings. Our conclusions align with long-term follow-up results from the ACTION trial, which suggested chemotherapy benefits in patients with non-optimal surgical staging, and with Kleppe et al., who noted no survival benefit from chemotherapy after lymph node dissection in early-stage ovarian cancer [30].

Oseledchyk et al. partially agreed with our third result, suggesting chemotherapy improves 5-year survival in those without lymphadenectomy, but no-lymphadenectomy itself may lead to occult metastasis [12]. Lymphadenectomy may reveal occult metastatic lesions and lead to a better prognosis for the patient [31]. A recent study further confirmed the necessity of chemotherapy for stage IC grade 3 patients after lymphadenectomy [32].

This retrospective study represents the largest SEER-based cohort investigating adjuvant chemotherapy in stage I OEC. A key strength lies in its alignment with current guidelines to address clinical controversies, supported by a robust sample size ensuring sufficient statistical power. We further enhanced validity through subgroup analyses and meta-analyses, incorporating our institutional experience.

However, limitations include potential selection bias from the lack of randomization, missing data on comorbidities, and unavailable disease-free, progression-free, disease-specific survival outcomes and not distinguishing between ‘none’ and ‘unknown’ lymphadenectomy status in SEER. Detailed chemotherapy regimen information was also lacking. Given the constraints of Kaplan-Meier analysis, future studies should incorporate additional covariates (e.g., chemotherapy cycles) and employ multifactorial Cox proportional hazards models for adjusted analyses. Furthermore, reliance on single-center data and limited comparable studies may restrict external validation and meta-analytic reliability. The studies included in the meta-analysis did not provide enough subtype-specific data for such comparisons, and we will take this as an important direction for future research.

### Implications for Practice and Future Research

The study suggests that adjuvant chemotherapy improves prognosis in stage IC grade 3 patients and may be considered for stage IC grade 1-2, especially without lymphadenectomy. Observation is suitable for stage IA/IB grade 1-2. Although no survival difference was found in stage IA/IB grade 3, guidelines and our experience support chemotherapy in this group. These findings aid in developing postoperative treatment protocols for stage I OEC, potentially improving outcomes.

## 5 Conclusion

The postoperative observation seemed to be preferable for patients with stage IA/IB grade 1-2. For patients with stage IA/IB grade 3, adjuvant chemotherapy was acceptable to be chosen as postoperative management. Adjuvant chemotherapy could enhance the prognosis of patients with stage IC, especially those patients without lymphadenectomy.

## Supplementary Materials





## Data Availability

The datasets generated and analysed during the current study are available from the corresponding author on reasonable request.

## Supplementary Materials

The supplementary material is available online at https://www.techscience.com/doi/10.32604/or.2025.065137/s1.
