# Supplementary material for: Adjuvant Chemotherapy Necessity in Stage I Ovarian Endometrioid Carcinoma: A SEER-Based Study Verified by Single-Center Data and Meta-Analysis
Source: Oncol Res. 2025 Sep 26;33(10):3007–22. doi: 10.32604/or.2025.065137 (PMC12493988; doi:10.32604/or.2025.065137)
Supplement: Supplementary file 1 [file OncolRes-33-65137-s001.docx]

**Supplementary Files:**

**Supplementary Table S1:** Binary logistic regression: predictors for the receipt of chemotherapy in OEC

| **Term** | | **OR** | **95% CI** | | ***p*-value** |
| --- | --- | --- | --- | --- | --- |
|  |  |  | **Lower** | **Upper** |  |
| **Age at diagnosis** | | 1.263 | 1.127 | 1.415 | **<0.001** |
| **Year of diagnosis** | | 1.201 | 1.097 | 1.315 | **<0.001** |
| **Race** | |  | | |  |
|  | White |  | *reference* |  | 0.329 |
|  | Black | 0.918 | 0.711 | 1.185 | 0.513 |
|  | Other | 1.129 | 0.938 | 1.359 | 0.198 |
| **Grade** | |  | | |  |
|  | G1 |  | *reference* |  | **<0.001** |
|  | G2 | 2.336 | 2.062 | 2.647 | **<0.001** |
|  | G3 | 4.563 | 3.860 | 5.393 | **<0.001** |
| **Substage** | |  | | |  |
|  | IA |  | *reference* |  | **<0.001** |
|  | IB | 1.565 | 1.236 | 1.981 | **<0.001** |
|  | IC | 4.091 | 3.629 | 4.612 | **<0.001** |
| **Lymphadenectomy** | | 1.148 | 1.006 | 1.311 | **0.040** |

Note: Binary logistic regression; *p*<0.05 is regarded as statistically significant (OR >1, chemotherapy administration more likely; OR <1, observation more likely).

**Supplementary Table S2:** Current guideline recommendations on whether to administer chemotherapy for early-stage OEC**.**

| Guidelines | **IA-G1** | **IA-G2** | **IA-G3** | **IB-G1** | **IB-G2** | **IB-G3** | **IC-G1** | **IC-G2** | **IC-G3** |
| --- | --- | --- | --- | --- | --- | --- | --- | --- | --- |
| **NCCN** (2024) [9] | O | O or C | C | O | O or C | C | O or C | C | C |
| **CSCO** (2024) [11] | O | O or C | C | O | O or C | C | C | C | C |
| **ESMO** (2023) [10] | O | C | C | O | C | C | O | C | C |
| **NCCN** (2022) [3] | O | O or C | C | O | O or C | C | O or C | C | C |
| **FIGO** (2021) [4] | O | O | O or C | O | O | O or C | C | C | C |
| **CSCO** (2021) [5] | O | O or C | C | O | O or C | C | C | C | C |
| **JSGO** (2020) [7] | O | C | C | O | C | C | C | C | C |
| **SEOM** (2020) [8] | O | O | C | O or C | O or C | C | O or C | O or C | C |
| **KSGO** (2018) [6] | O | O or C | C | O | O or C | C | C | C | C |

Note: O, observation; C, chemotherapy; NCCN, National Comprehensive Cancer Network; FIGO, International Federation of Gynecology and Obstetrics; CSCO, Chinese Society of Clinical Oncology; JSGO, Japan Society of Gynecologic Oncology; SEOM, Spanish Society for Medical Oncology; KSGO, Korean Society of Gynecologic Oncology; ESMO, European Society for Medical Oncology.

**Supplementary Table S3: The basic characteristics and quality evaluation of included studies.**

| **Study** | **Groups** | **Sample** | **Cancer type** | **Tumor grade** | **Sample size** | **Follow up** | **Endpoint** | **NOS score** |
| --- | --- | --- | --- | --- | --- | --- | --- | --- |
| Robert C. Young (1990) [16] | Adjuvant chemotherapy *vs* observation | Stage IA/B OC | S/M/E/C/O | G1-G2 | 38 *vs* 43 | More than 6 years | Recurrence | 8 |
| G. Bolis (1995) [17] | Adjuvant chemotherapy *vs* observation | Stage IA/B OC | S/M/E/C/O | G2-G3 | 44 *vs* 41 | 68 months | Recurrence | 7 |
| T. Paulsen (2011) [18] | Adjuvant chemotherapy *vs* observation | Stage IA/B OC | S/M/E/C/O | G1-G3 | 142 *vs* 130 | 56 months | Recurrence | 7 |
| Dai Shimizu (2015) [19] | Adjuvant chemotherapy *vs* observation | Stage IA OC  Stage IC OC | S/M/E/C/O | NA | 76 *vs* 17  38 *vs* 133 | 65 months | Recurrence | 7 |
| Masato Yoshihara (2021) [22] | Adjuvant chemotherapy *vs* observation | Stage IA OC  Stage IC OC | S/M/E/C/O | NA | 117 *vs* 131  31 *vs* 306 | More than 10 years | Recurrence | 7 |
| Brenna E Swift (2021) [20] | Adjuvant chemotherapy *vs* observation | Stage IA/B OC | E | G1-G2 | 66 *vs* 10 | 52 months | Recurrence | 7 |
| Caner Cakir (2021) [21] | Adjuvant chemotherapy *vs* observation | Stage IA OC | E | G1-G3 | 10 *vs* 12 | 92 months | Recurrence | 7 |

Note: OC, ovarian cancer; S, serous; M, mucinous; E, Endometrioid; C, clear cell; O, other; NA, not mentioned.
